# Decreased Mortality with Beta-Blocker Therapy in HFpEF Patients Associated with Atrial Fibrillation

**DOI:** 10.1155/2020/3059864

**Published:** 2020-05-13

**Authors:** Yanhua Yang, Suxia Guo, Ziyao Huang, Chunhua Deng, Lihua Chen, Guoxiang Zhou, Zhengwei Jian, Ruping He, Zhichao Huang, Yongzhao Yao, Jiongbin Lu, Zhiwen Hua, Yuli Huang

**Affiliations:** ^1^Department of Cardiology, Affiliated Dongguan People's Hospital, Southern Medical University, No. 3, South Wandao Road, Wanjiang District, Dongguan, Guangdong Province 523059, China; ^2^Department of Cardiology, Shunde Hospital, Southern Medical University (The First People's Hospital of Shunde), Foshan, China

## Abstract

**Background:**

There are no proven effective treatments that can reduce the mortality in heart failure with preserved ejection fraction (HFpEF), probably due to its heterogeneous nature which will weaken the effect of therapy in clinical studies. We evaluated the effect of beta-blocker treatment in HFpEF patients associated with atrial fibrillation (AF), which is a homogeneous syndrome and has seldom been discussed.

**Methods:**

This retrospective cohort study screened 955 patients diagnosed with AF and HFpEF. Patients with a range of underlying heart diseases or severe comorbidities were excluded; 191 patients were included and classified as with or without beta-blocker treatment at baseline. The primary outcome was all-cause mortality and rehospitalization due to heart failure. Kaplan-Meier curves and multivariable Cox proportional-hazards models were used to evaluate the differences in outcomes.

**Results:**

The mean follow-up was 49 months. After adjustment for multiple clinical risk factors and biomarkers for prognosis in heart failure, patients with beta-blocker treatment were associated with significantly lower all-cause mortality (hazard ratio (HR) = 0.405, 95% confidence interval (CI) = 0.233–0.701, *p*=0.001) compared with those without beta-blocker treatment. However, the risk of rehospitalization due to heart failure was increased in the beta-blocker treatment group (HR = 1.740, 95% CI = 1.085–2.789, *p*=0.022). There was no significant difference in all-cause rehospitalization between the two groups (HR = 1.137, 95% CI = 0.803–1.610, *p*=0.470).

**Conclusions:**

In HFpEF patients associated with AF, beta-blocker treatment is associated with significantly lower all-cause mortality, but it increased the risk of rehospitalization due to heart failure.

## 1. Introduction

Heart failure with preserved ejection fraction (HFpEF) is a difficult medical problem. Drugs that can improve the prognosis of heart failure with reduced ejection fraction (HFrEF), such as angiotensin-converting enzyme inhibitors, beta-blockers, spironolactone, and sacubitril/valsartan, have not been proven to have a protective effect on patients with HFpEF [1–5]. Evidence for beta-blockers in patients with HFpEF remains controversial [6, 7]. However, HFpEF is a highly heterogeneous clinical syndrome [8], with different diagnostic criteria among different studies. Even in the same study, selected subjects had different underlying heart diseases or comorbidities, which may be one of the underlying mechanisms for the heterogeneous effects of antiheart failure treatment [9, 10].

Atrial fibrillation (AF) is the most common arrhythmia in older people [11, 12]. AF combined with HFpEF, after excluding a range of underlying heart diseases and other comorbidities (e.g., malignant tumors, liver cirrhosis, hyperthyroidism, and severe renal disease), is a highly homogeneous group compared with all types of HFpEF. In these specific patients, AF is the initial or exacerbating factor of HFpEF [13–15], and the clinical features and prognostic factors might be different from those in other patients with HFpEF. Therefore, this study aimed to assess the effect of beta-blockers on mortality and heart failure rehospitalization in HFpEF patients associated with AF.

## 2. Materials and Methods

This study protocol retrospective cohort study was approved by the Ethics Committee of Shunde Hospital, Southern Medical University (20190903) and was consistent with Declaration of Helsinki. Written informed consent was obtained from all participants.

### 2.1. Study Population

A total of 955 patients were diagnosed with AF combined with HFpEF and discharged from January 2013 to May 2015. Among them, 206 were defined as HFpEF associated with AF and included in this study. The inclusion criteria included age ≤85 years and with documented AF with congestive heart failure (New York Heart Association functional class II or higher) with an ejection fraction (EF) ≥50%. Diagnosis of heart failure was according to the 2016 European Society of Cardiology guideline as follows: (1) symptoms typical of heart failure (HF) or signs typical of HF; (2) left ventricular ejection fraction (LVEF) ≥50%; (3) elevated levels of natriuretic peptides (BNP) ≥35 pg/ml; and (4) relevant structural heart disease (left ventricular hypertrophy/left atrial enlargement) and/or diastolic dysfunction [16].

We excluded patients with the following conditions: documented structural heart disease (including congenital heart disease, primary valve disease, or a history of cardiac valve replacement); pulmonary heart disease (chronic obstructive pulmonary disease, pulmonary embolism, and isolated right ventricular dysfunction caused by pulmonary disease); uncontrolled hyperthyroidism; documented cardiomyopathy (including ischemic cardiomyopathy, hypertrophic cardiomyopathy, dilated cardiomyopathy, arrhythmia, and right ventricular cardiomyopathy); severe renal insufficiency (glomerular filtration rate <30 ml/min/1.73 m^2^ or on dialysis); severe liver insufficiency (cirrhosis, severe hepatitis, or obstructive hepatobiliary disease); malignant tumors; severe systemic diseases; and acute myocardial infarction or those with acute stroke.

### 2.2. Laboratory and Echocardiographic Examinations

Biochemical blood measurements were determined using local standard laboratory procedures. The first results after admission, including hemoglobin, uric acid, albumin, BNP, and low-density lipoprotein cholesterol, were extracted from the hospital examination system. BNP levels were measured by the method of chemiluminescence (Beckmann DXI 800, USA). Conventional transthoracic echocardiography was used to measure LVEF within 3 days after admission using M-mode echocardiography (Philips IE33, The Netherlands).

### 2.3. Follow-Up and Outcome Assessment

All of the included patients were divided into those with or without beta-blocker treatment at discharge. The primary outcome was all-cause mortality, and secondary outcomes were all-cause rehospitalization and rehospitalization due to heart failure. All medical data were obtained through medical records, laboratory and ultrasonic systems, and telephone follow-up. Date collection was performed by trained staff, who were not informed of the purpose of this study. The patients who were loss to follow-up were not included for analysis.

### 2.4. Statistical Analysis

Categorical variables are shown as frequencies and percentages and were compared using the chi-square test. Nonnormally distributed continuous variables are shown as median (interquartile range) and the Mann–Whitney *U* test was used to compare these variables. Normally distributed continuous variables are shown as mean ± standard deviation (SD) and they were compared with Student's *t*-test. Survival curves were constructed for outcomes in patients with and without beta-blocker treatment. Multivariable Cox regression analysis was performed to calculate hazard ratios (HRs) and 95% confidence intervals (CIs) after adjusting for all potential confounders. All statistical analyses were performed using SPSS20 (IBM Corp., Asia Analytics, Shanghai, China). A *p* value < 0.05 was considered as statistical significance.

## 3. Results

### 3.1. Baseline Characteristics of the Patients

**Figure 1:**
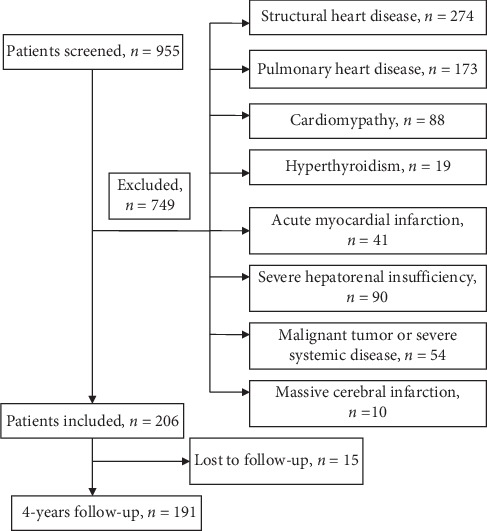
The flow diagram of screening, including, and follow-up. 955 patients were screened, of which 749 were excluded according to the exclusion criteria，206 patients were included in this study, 15 patients were lost to follow-up, and 191 patients were followed up by 49 months on the average.

After screening 955 hospitalized patients with AF combined with HFpEF, we excluded those with structural heart disease (*n* = 274), pulmonary heart diseases (*n* = 173), cardiomyopathy (*n* = 88), uncontrolled hyperthyroidism (*n* = 19), acute myocardial infarction (*n* = 41), severe hepatorenal insufficiency (*n* = 90), malignant tumors and systemic diseases (*n* = 54), and massive cerebral infarction (*n* = 10). Finally, 191 patients were defined as HFpEF associated with AF, and 78 had beta-blocker treatment (40.8%) at baseline (Figure 1). The baseline characteristics of patients with or without beta-blocker treatment are shown in Table 1. There were no significant differences in baseline data between the two groups. In the beta-blocker treatment group, 51 (65.4%) of them received bisoprolol (median (interquartile range) doses, 2.5 mg (1.25mg-3.75 mg)/d), and 27 (34.6%) received metoprolol (median (interquartile range) doses, 25 mg (23.75mg-47.5 mg)/d), respectively.

### 3.2. Outcome Data

**Figure 2:**
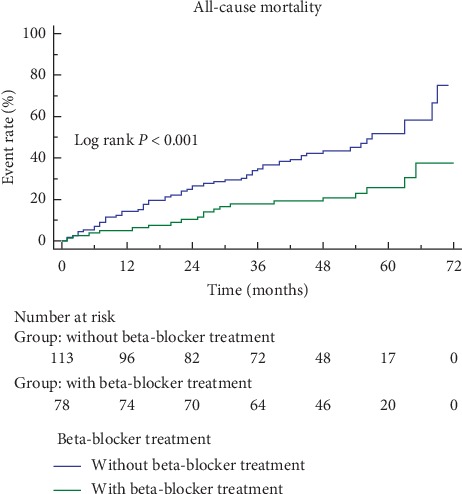
Kaplan-Meier curves for survival. Beta-blockers treatment group was associated with a significantly lower incidence of the all-cause death (log rank *p* < 0.001).

**Figure 3:**
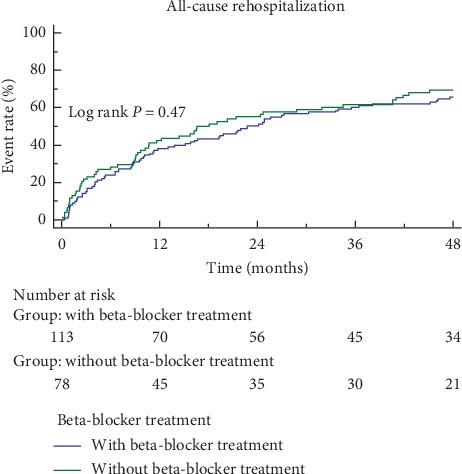
Kaplan-Meier curves for all-cause rehospitalization. There was no statistical difference in two groups analyzed by univariate cox regression model (log rank *p*=0.47).

**Figure 4:**
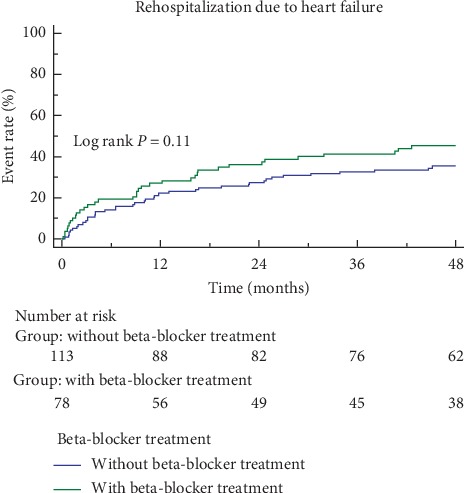
Kaplan-Meier curves for rehospitalization due to heart failure. There was no statistical difference in two groups analyzed by univariate cox regression model (log rank *p*=0.11).

The mean follow-up duration was 49 months. A total of 76 (39.8%) patients died during follow-up, and 56 (49.6%) did not have beta-blockers and 20 (25.6%) had beta-blockers. Corresponding survival curves are shown in Figure 2. Over the course of the study, 130 (68.1%) patients were rehospitalized, including 76 (58.5%) for worsening heart failure. As shown in Table 2, beta-blockers were associated with significantly lower mortality (HR = 0.405, 95% CI = 0.233–0.701, *p*=0.001), after adjusting by age, sex, smoke, stroke, hypertension, diabetes mellitus, history of acute myocardial infarction, heart rate, BNP level, and pulmonary artery pressure, which were commonly considered the factors to affect clinical outcomes, and also adjusting by diastolic blood pressure and albumin level, which were associated with all-cause mortality in univariate regression analysis. However, beta-blockers were not associated with all-cause rehospitalization (HR = 1.137, CI = 0.803–1.610, *p*=0.470) (Figure 3). The risk of rehospitalization due to HF was not significantly different between the two groups as analyzed by univariate analysis (log rank, *p*=0.11) (Figure 4). However, the risk of rehospitalization due to HF was increased in the beta-blocker treatment group (HR = 1.740, CI = 1.085–2.789, *p*=0.022) after adjusting by age, sex, smoke, stroke, hypertension, diabetes mellitus, history of acute myocardial infarction, pulmonary artery pressure, which were the known factors to affect HF rehospitalization, and also adjusting by BNP level and uric acid level, which were associated with HF rehospitalization in univariate regression analysis (Table 2).

## 4. Discussion

In this study, we found that beta-blocker treatment was associated with significantly lower mortality in HFpEF patients associated with AF. However, beta-blocker treatment appeared to slightly increase the risk of rehospitalization due to worsening HF.

AF is common in HF, with a reported prevalence of 21%–65% in HFpEF, which is higher than that reported in HFrEF (<10%–50%) [17]. The mechanism of HFpEF associated with AF may include the following: (1) In patients with HFpEF, the left atrial emptying fraction is significantly decreased [18]. Loss of atrial systole in AF impairs LV filling and can decrease cardiac output by up to 25%, particularly in patients with diastolic dysfunction [19]. Atrial contractile dysfunction is an important exacerbating factor of HFpEF. (2) In patients with prolonged AF, atrial remodeling, atrial size enlargement, valve ring dilation, failure of complete union of the two lobes, and secondary mitral regurgitation (MR) occur [20, 21]. In patients with HFpEF, left atrial fibrosis assessed by histology and magnetic resonance imaging accounts for 30.1% of the left atrial region [20]. This percentage is significantly higher than that of HFrEF (13%–27%) [22–24]. Therefore, AF is an important cause and aggravating factor in patients with HFpEF. (3) Irregular and/or rapid ventricular conduction in AF can lead to LV dysfunction and, in some patients, tachycardia-induced cardiomyopathy [19]. (4) Once AF causes obvious atrial enlargement and secondary MR, “MR leads to MR” may occur (i.e., MR leads to further atrial enlargement, leading to expansion of the valve ring and traction of the mitral valve to the apex, which in turn aggravates MR) [25–27]. (5) MR, AF, and HF are mutually causal, forming a vicious circle, which is the death triangle of patients with HFpEF [28, 29].

Currently, the evidence in support of beta-blocker use in patients with HFpEF remains controversial [6, 7, 30]. Most previous studies were on HFrEF with AF or HFpEF without AF, and there have not been any certain recommendations about beta-blockers in HFpEF combined with AF. A meta-analysis of individual patient data from 11 double-blind, randomized trials showed that beta-blockers were associated with a reduction in all-cause and cardiovascular mortality compared with placebo for patients in sinus rhythm. Beta-blockers were effective in all LVEF categories, except in the small subgroup where LVEF was >50%, but AF was not analyzed by stratification [31]. Another study showed that, in patients with HFpEF and a heart rate ≥70 bpm, high dose beta-blocker use was associated with a significantly lower risk of death [32]. HFpEF is a highly heterogeneous clinical syndrome, and diagnostic criteria varied in different studies, which reached inconsistent conclusions. In this study, we excluded patients with severe comorbidities. Therefore, all-cause mortality might more accurately describe the prognosis of these patients with HFpEF. We also excluded a range of underlying heart diseases to focus on HFpEF patients associated with AF. AF is a causal mechanism for worsening HFpEF. Therefore, targeting AF may be helpful in determining effective therapies for HFpEF [33, 34].

Beta-blockers have a variety of positive actions in HFpEF patients associated with AF as follows: (1) rate control and prevention of tachycardia-induced cardiomyopathy [33]; (2) blocking adrenergic receptors, which have direct effects on cardiomyocytes; and (3) alteration of vascular function and modification of the neuroendocrine response to heart failure [35]. The importance of these mechanisms may vary by etiology, LV phenotype, heart rhythm, and clinical indication [32].

In our study of HFpEF associated with AF, we found that use of beta-blockers in patients resulted in significantly lower mortality, but the risk of rehospitalization due to heart failure was increased. The possible pathophysiological mechanisms may be as follows: (1) beta-blockers can prolong diastolic period and increase ventricular volumes, therefore increasing the ventricular load [36]. Daniel et al. reported that beta-blocker use was associated with higher level of BNP in patients with HFpEF [37]. (2) Patients with HFpEF associated with AF are often combined with functional mitral regurgitation; therefore the EF would be overestimated. When the clinicians ignored this and the negative inotropic effect of beta-blocker, improper initial dose and titration may lead to worsening of heart failure.

According to these results, we recommend that beta-blockers should be used in HFpEF patients associated with AF. However, because beta-blockers can be negatively inotropic, there should be caution with the initial dosage and this should be gradually increased. Further prospective, randomized, controlled studies are required to confirm our conclusion.

There are some limitations in the current study. First, the duration of AF in patients was not provided in this study. Second, this was a retrospective clinical study, and the conclusion still needs to be confirmed by prospective, randomized, and controlled studies. Third, the oral anticoagulation therapy according to the guideline recommendation was greatly underused, maybe caused by concerning about the risk of bleeding in elderly patients. However, nowadays, no data have showed that whether there is an interactive effect of oral anticoagulation therapy and beta-blockers in patients with HF.

## 5. Conclusions

In HFpEF patients associated with AF, beta-blocker treatment is associated with significantly lower all-cause mortality, but not with all-cause rehospitalization. It should also be concerned that beta-blocker treatment may increase the risk of rehospitalization due to HF.

## Figures and Tables

**Table 1:** Baseline characteristic in HFpEF patients associated with AF.

	No beta-blocker (*n* = 113)	Beta-blocker (*n* = 78)	All patients (*n* = 191)	*p* value
Age, years	77.0 (72.5–80.0)	76.0 (69.0–80.3)	77 (71–80)	0.12
Male, *n* (%)	58 (51.3)	35 (44.9)	93 (48.7)	0.380
Current or past smoker, *n* (%)	29 (25.7)	14 (17.9)	43 (22.5)	0.210
Alcoholic, *n* (%)	6 (5.3)	2 (2.6)	8 (4.2)	0.475
Hypertension, *n* (%)	80 (70.8)	47 (60.3)	127 (66.5)	0.129
Diabetes mellitus, *n* (%)	29 (25.7)	28 (35.9)	57 (29.8)	0.129
History of AMI, *n* (%)	9 (8.0)	9 (11.5)	18 (9.4)	0.406
History of stroke, *n* (%)	33 (29.2)	16 (20.5)	49 (25.7)	0.176
AECI, *n* (%)	12 (10.6)	11 (14.1)	23 (12.0)	0.467
ARB, *n* (%)	34 (30.1)	18 (23.1)	52 (27.2)	0.285
Digoxin, *n* (%)	44 (38.9)	31 (39.7)	75 (39.3)	0.911
Oral anticoagulant, *n* (%)	51 (45.1)	41 (52.6)	92 (48.2)	0.312
Statin, *n* (%)	73 (64.6)	45 (57.7)	118 (61.8)	0.334
Non-dihydropyridine calcium ion antagonist	5 (4.4)	3 (3.8)	8 (4.2)	1.000
Heart rate (beats/min)	80.0 (75.5–90.0)	78.0 (74.0–85.3)	80 (75–88)	0.206
Systolic blood pressure (mmHg)	125.3 ± 16.9	122.9 ± 17.2	124.3 ± 17.0	0.334
Diastolic blood pressure (mmHg)	76.0 (66.0–83.5)	75.5 (66.0–80.0)	76 (66–80)	0.352
Hemoglobin (g/L)	120.0 (109.0–132.5)	122.5 (115.0–137.0)	122 (111–134)	0.295
Uric acid (umol/L)	400.8 ± 136.6	392.7 ± 138.3	397.5 ± 137.0	0.687
Albumin (g/L)	37.3 ± 4.7	38.3 ± 3.7	37.7 ± 4.3	0.114
BNP (pg/ml)	279.0 (169.1–439.5)	232.9 (181.1–495.0)	275.0 (176.8–449.0)	0.783
LDL-c (mmol/L)	2.62 (1.94–3.24)	2.68 (2.00–3.26)	2.63 (1.94–3.25)	0.965
Left atrial diameter (mm)	44 (40–48)	44 (41–49)	44 (40–48)	0.769
Right atrial diameter (mm)	42 (36–47)	41.5 (36–48)	42 (36–47)	0.688
LVEDD (mm)	47 (43–50.5)	46.5 (44–51)	47 (43–51)	0.762
Pulmonary artery pressure (mmHg)	42.5 ± 12.9	41.8 ± 11.1	42.2 ± 12.2	0.709

Continuous variables are presented as median (interquartile range) or mean (standard deviation). Categorical variables are expressed as number (percentages). AF, atrial fibrillation; AMI, acute myocardial infarction; ACEI, angiotensin-converting enzyme inhibitor; ARB, angiotensin receptor blocker; BNP, brain natriuretic peptide; HFpEF, heart failure with preserved ejection fraction; LDL-c, low-density lipoprotein cholesterol; LVEDD, left ventricular end-diastolic dimension.

**Table 2:** Outcomes in patients of HFpEF associated with AF with or without beta-blocker treatment.

	Without beta-blocker	With beta-blocker	Unadjusted		After adjusted	
	(*n* = 113)	(*n* = 78)	HR (95%CI)	*p* Value	HR (95%CI)	*p* value
All-cause mortality	56 (49.6%)	20 (25.6%)	0.422 (0.253–0.704)	0.001	0.405 (0.233–0.701)^#^	0.001^#^
All-cause rehospitalization	75 (66.4%)	55 (70.5%)	1.137 (0.803–1.610)	0.470	1.200 (0.824–1.747)^*∗*^	0.342^*∗*^
HF rehospitalization	40 (35.4%)	36 (46.2%)	1.441 (0.918–2.260)	0.112	1.740 (1.085–2.789)^*∗*^	0.022^*∗*^

AF, atrial fibrillation; CI, confidence interval; HF, heart failure; HR, hazard ratio; HFpEF, heart failure with preserved ejection fraction. ^#^Adjusted by age, sex, smoke, stroke, hypertension, diabetes mellitus, history of acute myocardial infarction, heart rate, brain natriuretic peptide (BNP) level, and pulmonary artery pressure, which were commonly considered the factors to affect clinical outcomes, and also adjusted by diastolic blood pressure and albumin level, which were associated with all-cause mortality in univariate regression analysis. ^*∗*^Adjusted by age, sex, smoke, stroke, hypertension, diabetes mellitus, history of acute myocardial infarction, and pulmonary artery pressure, which were the known factors to affect HF rehospitalization, and also adjusted by BNP level and uric acid level, which were associated with HF rehospitalization in univariate regression analysis.

## Data Availability

The data used to support the findings of this study are available from the corresponding author upon request.

## References

[1] Maggioni A. P., Anker S. D., Dahlström U. (2013). Are hospitalized or ambulatory patients with heart failure treated in accordance with european society of cardiology guidelines? Evidence from 12 440 patients of the esc heart failure long-term registry. *European Journal of Heart Failure*.

[2] Filippatos J. G. F., Tendera M., Adamus J. (2006). The perindopril in elderly people with chronic heart failure (PEP-CHF) study. *European Heart Journal*.

[3] Pitt B., Pfeffer M. A., Assmann S. F. (2014). Spironolactone for heart failure with preserved ejection fraction. *New England Journal of Medicine*.

[4] Massie B. M., Carson P. E., McMurray J. J. (2008). Irbesartan in patients with heart failure and preserved ejection fraction. *New England Journal of Medicine*.

[5] King J. B., Bress A. P., Reese A. D., Munger M. A. (2015). Neprilysin inhibition in heart failure with reduced ejection fraction: a clinical review. *Pharmacotherapy: The Journal of Human Pharmacology and Drug Therapy*.

[6] Tsujimoto T., Kajio H. (2018). Beta-blocker use and cardiovascular event risk in patients with heart failure with preserved ejection fraction. *Sci Rep*.

[7] Bavishi C., Chatterjee S., Ather S., Patel D., Messerli F. H. (2015). Beta-blockers in heart failure with preserved ejection fraction: a meta-analysis. *Heart Failure Reviews*.

[8] Ferrari R., Böhm M., Cleland J. G. F. (2015). Heart failure with preserved ejection fraction: uncertainties and dilemmas. *European Journal of Heart Failure*.

[9] Sanjiv J., Daniel H., Rahul C. (2014). Phenotypic spectrum of heart failure with preserved ejection fraction. *Heart Fail Clin*.

[10] Shah A. M., Solomon S. D. (2012). Phenotypic and pathophysiological heterogeneity in heart failure with preserved ejection fraction. *European Heart Journal*.

[11] Nabil N., Mirza D., Azra D. (2017). The impact of risk factors and comorbidities on the incidence of atrial fibrillation. *Mater Sociomed*.

[12] Townsend N., Wilson L., Bhatnagar P., Wickramasinghe K., Rayner M., Nichols M. (2016). Cardiovascular disease in Europe: epidemiological update 2016. *European Heart Journal*.

[13] Goyal P., Almarzooq Z. I., Cheung J. (2018). Atrial fibrillation and heart failure with preserved ejection fraction: insights on a unique clinical phenotype from a nationally-representative United States cohort. *International Journal of Cardiology*.

[14] Kono T., Sabbah H. N., Rosman H., Alam M., Stein P. D., Goldstein S. (1992). Left atrial contribution to ventricular filling during the course of evolving heart failure. *Circulation*.

[15] Kotecha D., Lam C. S. P., Van Veldhuisen D. J., Van Gelder I. C., Voors A. A., Rienstra M. (2016). Heart failure with preserved ejection fraction and atrial fibrillation. *Journal of the American College of Cardiology*.

[16] Ponikowski P., Voors A. A., Anker S. D. (2016). 2016 ESC Guidelines for the diagnosis and treatment of acute and chronic heart failure. *European Heart Journal*.

[17] Sartipy U., Dahlström U., Fu M., Lund L. H. (2017). Atrial fibrillation in heart failure with preserved, mid-range, and reduced ejection fraction. *JACC: Heart Failure*.

[18] Vojtech M., Seok-Jae H. (2015). Left atrial remodeling and function in advanced heart failure with preserved or reduced ejection fraction. *Circulation Heart Fail*.

[19] Kotecha D., Piccini J. P. (2015). Controversies in cardiovascular medicine: atrial fibrillation in heart failure: what should we do?. *European Heart Journal*.

[20] Felix H., Daniel M., David B. (2018). Atrial remodelling in heart failure: recent developments and relevance for heart failure with preserved ejection fraction. *ESC Heart Failure*.

[21] Delgado V., Bax J. J. (2017). Atrial functional mitral regurgitation from mitral annulus dilatation to insufficient leaflet remodeling. *Circ Cardiovasc Imaging*.

[22] Kihara T., Gillinov A. M., Takasaki K. (2009). Mitral regurgitation associated with mitral annular dilation in patients with lone atrial fibrillation: an echocardiographic study. *Echocardiography*.

[23] Gertz Z. M., Raina A., Saghy L. (2011). Evidence of atrial functional mitral regurgitation due to atrial fibrillation. *Journal of the American College of Cardiology*.

[24] Kagiyama N., Hayashida A., Toki M. (2017). Insufficient leaflet remodeling in patients with atrial fibrillation: association with the severity of mitral regurgitation. *Circ Cardiovasc Imaging*.

[25] Sanders N. A., Supiano M. A., Lewis E. F. (2018). The frailty syndrome and outcomes in the TOPCAT trial. *European Journal of Heart Failure*.

[26] Silbiger J. J. (2014). Does left atrial enlargement contribute to mitral leaflet tethering in patients with functional mitral regurgitation? Proposed role of atriogenic leaflet tethering. *Echocardiography*.

[27] Tang Z., Fan Y.-T., Wang Y., Jin C.-N., Kwok K.-W., Lee A. P.-W. (2019). Mitral annular and left ventricular dynamics in atrial functional mitral regurgitation: a three-dimensional and speckle-tracking echocardiographic study. *Journal of the American Society of Echocardiography*.

[28] Noack T., Kiefer P., Mallon L. (2019). Changes in dynamic mitral valve geometry during percutaneous edge-edge mitral valve repair with the MitraClip system. *Journal of Echocardiography*.

[29] Schrage B., Kalbacher D., Schwarzl M. (2018). Distinct hemodynamic changes after interventional mitral valve edge-to-edge repair in different phenotypes of heart failure: an integrated hemodynamic analysis. *Journal of the American Heart Association*.

[30] Yura M., John G., Cleland F. (2015). Should beta-blocker be used in patients with heart failure and Atrial fibrillation?. *Clinical Therapeutics*.

[31] John G. F., Karina V., Marcus D. (2018). Beta-blockers for heart failure with reduced, mid-range, and preserved ejection fraction: an individual patient-level analysis of double-blind randomized trials. *European Heart Journal*.

[32] Lam P. H., Gupta N., Dooley D. J. (2018). Role of high-dose beta-blockers in patients with heart failure with preserved ejection fraction and elevated heart rate. *The American Journal of Medicine*.

[33] Piccini J. P., Allen L. A. (2017). Heart failure complicated by atrial fibrillation. *JACC: Heart Failure*.

[34] Senni M., Paulus W. J., Gavazzi A. (2014). New strategies for heart failure with preserved ejection fraction: the importance of targeted therapies for heart failure phenotypes. *European Heart Journal*.

[35] von Lueder T. G., Kotecha D., Atar D. (2017). Neurohormonal blockade in heart failure. *Cardiac Failure Review*.

[36] Daniel N. S., Timothy B. P., Margaret I. (2019). Association of beta-blocker use with heart failure hospitalizations and cardiovascular disease mortality among patients with heart failure with a preserved ejection fraction. A secondary analysis of the TOPCAT trial. *JAMA Network Open*.

[37] Daniel N. S., Timothy B. P., Margaret I. (2020). Are beta-blockers associated with increased natriuretic peptide levels in HFpEF? Insights from the TOPCAT trial. *Journal of the American College of Cardiology*.

